# Supplementary material for: Protein–Protein Interfaces: A Graph Neural Network Approach
Source: Int J Mol Sci. 2024 May 28;25(11):5870. doi: 10.3390/ijms25115870 (PMC11173158; doi:10.3390/ijms25115870)
Supplement: Supplementary file 1 [file ijms-25-05870-s001.zip › ijms-2988191-supplementary.pdf]

Supplementary Materials

May 15, 2024

Features Description

Each residue is described by a collection of features, gathered into a vector $\mathbf{x} \in R^{89}$. In detail, this includes the description of the residue position in the three–dimensional space, *i.e.*, *x, y,* and *z* coordinates, the *accessible surface area*, the residue torsion angles ϕ and ψ and the *temperature factor*. The following features belong to DSSP descriptors, which characterize protein secondary structures by referring to 8 SSE standard classes: *α–helix (4–12)*, *isolated β–bridge* residue, *strand*, *α–helix (3–10)*, *π–helix*, *turn*, *bend*, and "none" whether the residue is not part of a regular secondary structure. The set comprises also two indicators of the energy associated with a hydrogen bond between the NH group of an amino acid residue and the oxygen atom of another molecule, and two other refer to the bond between the oxygen atom of the residue and the NH group of another molecule. Additionally, the DSSP attributes include two features referred to the number of hydrogen bond *donor* and *acceptor* atoms (typically nitrogen or oxygen) in a molecule or a specific region of a protein. Next, the Meiler representation of the specific residue is reported. It consists in an intrinsic collection of geometric shape indicators approximating backbone and side chain atoms, which reduces the complexity of the structural description. These features include *steric parameter*, *polarizability*, *volume*, *hydrophobicity*, *isoelectric point*, *helix* and *sheet probability.* The last and most considerable subset of features refers to the ExPASy service, which provides general non–residue specific amino acid descriptors. It comprises the *isoelectric points* and the *molecular weight* of the protein, the *number codons* encoding for a specific amino acid, and the *relative mutability*, that is a measure of the relative mutation rate of the corresponding amino acid. Additionally, the set includes the following:

- *pKa COOH α, pKa NH3, pka R–group*: the pKa of the α–carboxyl, amino (NH3+) and R–group of an amino acid;
- *hydrophobicity janin, miyazawa, argos, roseman, tanford, wolfenden, welling, wilson, parker, chothia, rose, eisenberg, sweet, woods, doolittle, manavalan, leo, black, breese, fauchere, guy, pH 3–4, pH 7–5, mobility, and high–performance liquid chromatography FBA, TFA, 2–1, 7–4*: these are differ-

ent hydrophobicity scales or indices, each calculated using different methods or algorithms to measure the hydrophobicity of amino acids;

- *β–turn fasman, α–helix fasman, β– sheet fasman*: secondary structure prediction scores calculated using the Fasman method;
- *β–turn roux, α–helix roux, β–sheet roux, coil roux*: secondary structure prediction scores calculated using the Roux method;
- *α–helix levitt, β–sheet levitt, β–turn levitt*: secondary structure prediction scores calculated using the Levitt method;
- *totalβ–strand, antiparallelβ–strand, parallelβ–strand*: measures related to β–strands in proteins;
- *polarity zimmerman, polarity grantham*: measures of the polarity of an amino acid side chain;
- *average buried, average flexibility*: measures related to the burial or flexibility of amino acid residues.
- *amino acid composition, amino acid swiss–prot*: amino acid composition of a protein.

Finally, the ExPASy subset provides *refractivity*, *i.e.* a measure of the ability of an amino acid side chain to bend light, *recognition factors* affecting protein–protein or protein–ligand, the *ratio side* of polar to non-polar side chains and *bulkiness*, that is a measure of the steric bulk of an amino acid side chain.

Eleven features were considered for the edges to describe the bond between residues, such as the *distance*. A one–hot encoding was used to identify the bond type through the following features:

- *aromatic*: indicates whether an amino acid residue contains an aromatic ring structure, such as phenylalanine, tyrosine, or tryptophan;
- *backbone carbonyl*: indicates the presence or involvement of a backbone carbonyl group in a protein structure. This feature may indicate the formation of secondary structures like beta sheets or the participation in hydrogen bonding interactions;
- *disulfide*: indicates the presence of a disulfide bond between two cysteine residues in a protein. Disulfide bonds contribute to the stabilization of protein structure;
- *H bond*: the presence or involvement of hydrogen bonds in a protein structure. Hydrogen bonds form between electronegative atoms, such as oxygen or nitrogen, and hydrogen atoms covalently bonded to other electronegative atoms;

- *hydrophobic*: indicates whether an amino acid residue is hydrophobic, meaning it tends to avoid contact with water and may be found buried within the protein core;
- *ionic*: indicates the presence or involvement of ionic interactions between charged amino acid residues in a protein. These interactions occur between oppositely charged side chains, such as those of lysine (positive) and aspartate (negative);
- *peptide*: indicates the presence of peptide bonds in a protein structure, which link amino acid residues together to form the protein chain;
- *salt bridge*: a type of ionic interaction between positively and negatively charged amino acid residues that are typically distant from each other in the protein sequence but come into close proximity in the folded protein structure, stabilizing the protein;
- *Van der Waals*: represents van der Waals interactions, which are weak attractive forces between atoms or molecules due to fluctuating electron distributions. These interactions contribute to the overall stability of protein structures;
- *Van der Waals clash*: indicates steric clashes or overlaps between atoms in a protein structure that violate van der Waals radii constraints. These clashes may indicate structural problems or clashes that need to be resolved during structure refinement.
